# RetroSeeker reveals the characteristics, expression, and evolution of a large set of novel retrotransposons

**DOI:** 10.1007/s44307-023-00005-5

**Published:** 2023-10-31

**Authors:** Junhong Huang, Zhirong Chen, Bin Li, Lianghu Qu, Jianhua Yang

**Affiliations:** 1 MOE Key Laboratory of Gene Function and Regulation, State Key Laboratory of Biocontrol, School of Life Sciences, Sun Yat-Sen University, Guangzhou, 510275 Guangdong China; 2 The Fifth Affiliated Hospital, Sun Yat-Sen University, Zhuhai, 519000 Guangdong China

**Keywords:** Retrotransposon, Expression, New gene, Tissue specific, Evolution

## Abstract

Retrotransposons are highly prevalent in most animals and account for more than 35% of the human genome. However, the prevalence, biogenesis mechanism and function of retrotransposons remain largely unknown. Here, we developed retroSeeker, a novel computational software that identifies novel retrotransposons from pairwise alignments of genomes and decodes their biogenesis, expression, evolution and potential functions. We discovered that the majority of new retrotransposons exhibit a specific L1 endonuclease cleavage motif, with some motifs precisely located ten nucleotides upstream of the insertion site. We identified that a large number of candidate functional genes might be generated through a retrotransposition mechanism. Importantly, we uncovered previously uncharacterized classes of retrotransposons related to histone genes, mitochondrial genes and vault RNAs. Moreover, we elucidated the tissue-specific expression of retrotransposons and demonstrated their ubiquitous expression in various cancer types. We also revealed the complex evolutionary patterns of retrotransposons and identified numerous species-specific retrotransposition events. Taken together, our findings establish a paradigm for discovering novel classes of retrotransposons and elucidating their new characteristics in any species.

**Supplementary Information:**

The online version contains supplementary material available at 10.1007/s44307-023-00005-5.

## Introduction

Retrotransposons are the mobile elements with the highest prevalence in the genomes of most animals, accounting for approximately 38% of human DNA (Kazazian and Moran 2017; Wells and Feschotte 2020; Fueyo et al. 2022). Retrotransposons include autonomous long interspersed elements (LINEs, represented by LINE-1) and nonautonomous short interspersed elements (SINEs, represented by Alu and MIR), which require LINE-derived proteins for retrotransposition (Moran et al. 1996; Cordaux and Batzer 2009). The primary mechanism of retrotransposition is referred to as target-primed reverse transcription (TPRT) (Dewannieux et al. 2003), commonly referred to as the “copy and paste” mechanism (Gilbert et al. 2002). In brief, the LINE-derived protein ORF1 cleaves the first strand of DNA at the target site, which is often marked by the TTAAAA motif, while simultaneously capturing the poly(A) tail of the transcribed RNA (Gilbert et al. 2002; Dewannieux et al. 2003). Simultaneously, another protein, ORF2, generates complementary DNA (cDNA) from the retrotransposon transcript, utilizing cleaved genomic DNA as a primer (“copy” process) (Gilbert et al. 2002; Dewannieux et al. 2003). Subsequently, the second strand of the target DNA is cleaved, and the second strand of the retrotransposon is synthesized (“paste” process) (Gilbert et al. 2002; Dewannieux et al. 2003). This series of events results in target site duplications (TSDs) that flank the retrotransposons (Gilbert et al. 2002; Dewannieux et al. 2003). Thus, retrotransposons can be distinguished from other genomic rearrangements by the presence of two characteristic features: TSDs and 3’-poly(A) signals flanking retrotransposons. However, due to the predominantly random integration of retrotransposons into the host genome (Fujiwara 2015), preferences for genome insertion and their impact on shaping the genome remain largely elusive.

LINE-derived proteins also facilitate the retrotransposition of protein-coding RNAs (Maestre et al. 1995) and some types of noncoding RNAs (ncRNAs), such as small nucleolar RNA (snoRNA) (Weber 2006), small nuclear RNA (snRNA) (Doucet et al. 2015), and Y RNA (Perreault et al. 2005). The retrotransposition generates new functional RNA copies, which can recover the roles of their parental genes when the latter are inactivated by mutations, which is exemplified by the mouse ACA36 snoRNA located within the intron of the DKC1 gene (Weber 2006). Furthermore, the increased sequence diversity exhibited by various retrotransposition-active ncRNAs, compared to classical LINE and SINE elements, makes them valuable markers for studying the evolution of vertebrate genomes (Weber 2006). Nevertheless, prior investigations have predominantly relied on homology searches and manual delineation of one type of retrotransposition-active ncRNA, so comprehensive and systematic exploration is lacking. Importantly, it is yet to be determined whether other varieties of ncRNAs act as sources for retrotransposition and undergo reverse transcription through the LINE-1 retrotransposon machinery.

While most retrotransposition events are harmful or neutral, they can provide raw material for creating new functional genes and have emerged as major sources of evolutionary novelties. They act as gene promoters, influence alternative splicing of genes, induce sequence alterations in existing genes, and even contribute to the de novo origination of genes (Kaessmann et al. 2009). Compared to processed pseudogenes that are also generated by retrotransposition, newly functional genes have a profound impact on the evolution of physiological, morphological, behavioural, and reproductive phenotypic traits (Carelli et al. 2016). They are believed to play a significant role in driving brain evolution in mammals (Ferrari et al. 2021). However, due to the absence of an appropriate genome-scale analysis methodology, the specific retrotransposition events responsible for generating new genes and the actual number of new genes generated by retrotransposition remain unclear.

In this study, we utilized pairwise alignments of genomes from various species to trace the hidden “copy and paste” history of retrotransposition and developed a computational tool called retroSeeker, which facilitates comprehensive exploration for retrotransposons. Our study reveals the recurrent presence of specific motifs in proximity to retrotransposons. Furthermore, our findings indicate that retrotransposons contribute to the emergence of new genes through two primary mechanisms: integration within intragenic regions and integration within intergenic regions. Notably, we discovered novel classes of retrotransposons related to histone genes, the mitochondrial genome and vault RNAs. Interestingly, many retrotransposons showed specific expression patterns in certain tissues but exhibited ubiquitous expression in various types of cancers. Furthermore, we successfully identified numerous retrotransposons unique to each species, showcasing intricate evolutionary trajectories. Together, our results reveal the potential biogenesis mechanisms, expression, and evolution of a large set of novel retrotransposons.

### Main

#### A novel computational approach designed to discover retrotransposons

**Fig. 1:**
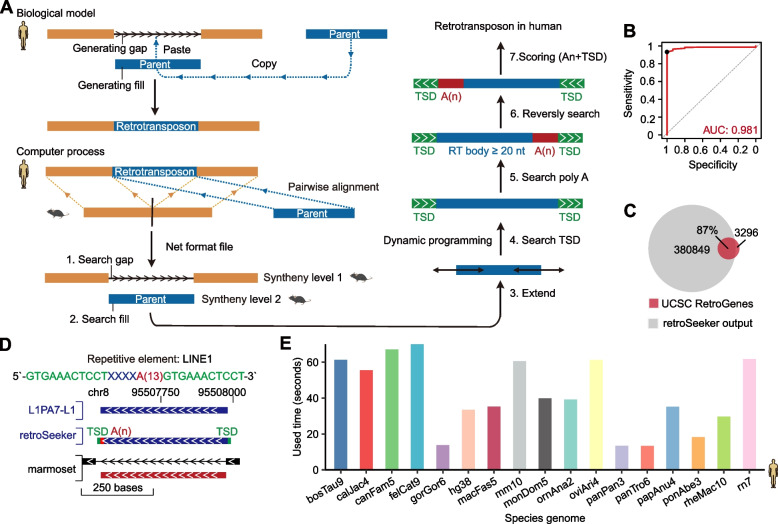
New computational approach for the discovery of retrotransposons. **A** Schematic depiction of retroSeeker arithmetic and workflow. Left panel: characterization of the net file format. The net-format file is derived from the pairwise alignment of two whole genomes (e.g., mouse query to human reference). The alignment will produce two results, gap and fill, where “gap” indicates that the region exists in the reference genome but not in the query genome and “fill” indicates that the region exists in both the reference and query genomes. The human–mouse alignment net-format file shows the alignment of the syntenic portions of the human and mouse genomes. Levels 1 and 2 are two synteny levels: Level 1 corresponds to the mouse orthologue of the host gene of the human retrotransposon, and Level 2 corresponds to the mouse orthologue of the human retrotransposon. Right panel: characterization of the fill region of level 2 and determination of the retrotransposons. Examination of the human sequence enables recognition of the polyA tail A(n) (red region) and the TSD (green region) and precise localization of the retrotransposon insertion point between two TSDs. **B** Receiver operating characteristic curve of the retrotransposon score calculated by retroSeeker; area under the curve (AUC) values are shown. **C** Venn diagram depicting the number of unique and shared items that were identified by retroSeeker and UCSC RetroGenes. **D** Genome browser visualization for retrotransposons. The whole sequence of the retrotransposon on the top is shown in various colours; green represents TSDs at both termini, blue represents the gene body, and red represents poly(A), of which the number of nucleotides in the poly(A) region is shown in brackets, that is, A(n). The first track shows the information of the known gene annotation. The second track shows the identification result from retroSeeker. The third track partly shows the input net files for retroSeeker. **E** The time taken by retroSeeker for different input net files. The genome versions of the net files are shown

To comprehensively search for retrotransposons, we developed a computational tool called retroSeeker that uses net files, pairwise alignments of two different genomes generated by the lastz aligner and UCSC utilities (see Methods), to discover the copy and paste events of retrotransposition. In the net files, the parental gene generated a "fill" region, and copy and paste retrotransposition generated a "gap" region within the new genomic locus (Weber 2006) (Fig. 1A). The retroSeeker tool initiates the process by conducting pairwise genome alignments between two different species (Fig. 1A) to generate the corresponding net files. Subsequently, it scans the hidden gap and fill regions stemming from retrotransposition occurrences identified within these net files. Once a potential retrotransposition region was identified, we employed a dynamic programming algorithm to search and score the flanking TSD and poly(A) sequences (Methods).

To evaluate the sensitivity and specificity of retroSeeker, we carried out a series of simulation studies. We randomly inserted a set of LINE1s into a simulated genome multiple times to generate genomes containing these new retrotransposons (Methods). We found that the putative retrotransposons were recalled with high specificity (~ 1.00) and sensitivity (0.93, Fig. 1B). Significantly, through retroSeeker analysis, a total of 380,849 potential retrogenes were uncovered within the human genome. This encompasses approximately 87% of the retrogenes recognized by the UCSC Genome Browser and achieves a remarkable 14-fold expansion of the retrotransposon catalogue (Fig. 1C). For example, retroSeeker successfully identified the presence of an FKBP8 retrotransposon inserted within the intron of PPP1R12B, consistent with the findings in the UCSC Genome Browser (Figure S1A). In addition, we identified several well-known varieties of retrotransposons originating from repetitive elements, such as LINE1 (Fig. 1D), Alu (Figure S1B) and SVA (Figure S1C) retrotransposons. Furthermore, retroSeeker demonstrated its capability to unveil retrogenes originating from various types of ncRNAs. Notably, 7SL RNA (Figure S1D), snoRNA (Figure S1E), snRNA (Figure S1F), and Y RNA (Figure S1G) were all successfully identified, each exhibiting perfect TSDs and canonical poly(A) regions.

To evaluate the running speed of retroSeeker, we used pairwise alignment data sourced from diverse species. Remarkably, the majority of our analyses were successfully executed in a minute, with the swiftest completion time an astonishingly brief 1.4 s (Fig. 1E, Figure S1H and I). Together, these results suggest that our retroSeeker approach not only shows high sensitivity, specificity and efficiency in discovering various retrotransposons but can also uncover a large number of novel retrogenes.

#### Distinctive characteristics of retrotransposons across diverse evolutionary clades

**Fig. 2:**
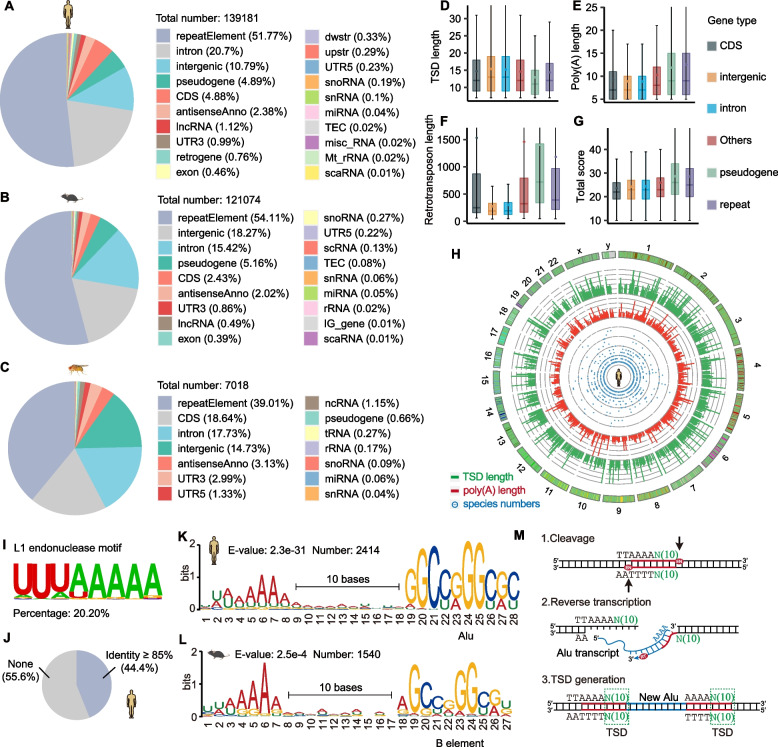
Characteristics of retrotransposons. **A** to **C** Distribution of identified retrotransposons in humans (**A**), mice (**B**) and flies (**C**) in annotated gene types. **D** to **G** Box plot showing the values of TSD length (**D**), polyA length (**E**), retrotransposon length (**F**) and score (**G**) of the identified retrotransposon. Each box shows the first quartile, median, and third quartile. H, Circos plot illustrating the TSD length, poly(A) length and species numbers of identified retrotransposons in humans. The plot legend from the outer circle to the inner circle is shown. **I** Sequence motif obtained within the upstream and downstream 20 nt sequences of 5ʹ-start sites of the human retrotransposons. **J** Pie plots displaying the percentages of L1 endonuclease cleavage motifs (TTAAAA) identified within human retrotransposons. **K** and **L** Sequence motifs obtained within the upstream and downstream 20 nt sequences of 5ʹ-start sites of the retrotransposons in humans (**K**) and mice (**L**). **M** Schematic depiction of second-strand cleavage specificity during target-site primed reverse transcription (TPRT) events

To systematically investigate the characteristics of retrotransposons, we applied retroSeeker to humans, mice and flies (Table S1) with stringent criteria requiring a TSD length of ≥ 7 and a poly(A) length of ≥ 5 (Methods). We identified 139,181, 121,074 and 7018 retrotransposons from humans, mice and flies, respectively. Notably, retrotransposons were primarily located in repetitive elements (51%), introns (20%) and intergenic regions (10%) throughout the human genome (Fig. 2A and Table S2). The majority of retrotransposons associated with repetitive elements were mapped to primate-specific Alu elements, LINE 1, and endogenous retroviruses (ERVs, Figure S2A). In mice, a comparable distribution pattern of genomic regions was observed (Fig. 2B and Table S3), and the majority of retrotransposons associated with repetitive elements were found to map primarily to rodent-specific B elements, including B1 (15%), B2 (10%), and B4 (3%) (Figure S2B). Interestingly, in flies, a notably higher percentage of retrotransposons (18%) was found within protein-coding sequences (Fig. 2C and Table S4). Furthermore, a predominant fraction of these retrotransposons was associated with repetitive elements, specifically Gypsy (27%), Jockey (7%), and Pao (4%) (Figure S2C).

To uncover the structural features of retrotransposons, we examined the lengths and scores of their individual components. Our analysis revealed a TSD length spanning from 7 to 18 nucleotides (nt, Fig. 2D), a poly(A) length ranging from 6 to 13 nt (Fig. 2E), a whole retrotransposon length spanning from 100 to 1500 nt (Fig. 2F), and a total score ranging from 20 to 30 (Fig. 2G). However, our investigation did not discover any correlation between TSD and poly(A) length for each retrotransposon (Fig. 2H, Figure S2D, and E).

To investigate whether there are specific sequence elements within retrotransposons, we de novo-identified motifs within the 20 nucleotides upstream or downstream of the 5'-start sites. Strikingly, we found that approximately 20.2% of retrotransposons possessed the L1 endonuclease cleavage motif (Fig. 2I). Furthermore, 44.4% of retrotransposons had motifs similar to the L1 endonuclease cleavage motif (Fig. 2J). To investigate the locus specificity of L1 endonuclease activity, we calculated the distance between the motif and the inserted retrotransposon. Interestingly, we discovered a subset of retrotransposons that contained sharp Alu elements (Fig. 2K) or B elements (Fig. 2L) located precisely 10 nucleotides downstream of the L1 endonuclease cleavage motif, suggesting that second-strand cleavage occurs at precise locations during TPRT events (Fig. 2M). Notably, the region adjacent to the 5'-TSD tended to contain U-rich (Figure S2F) or A-rich elements (Figure S2G).

#### Retrotransposition generate new genes and facilitate functional evolution

**Fig. 3:**
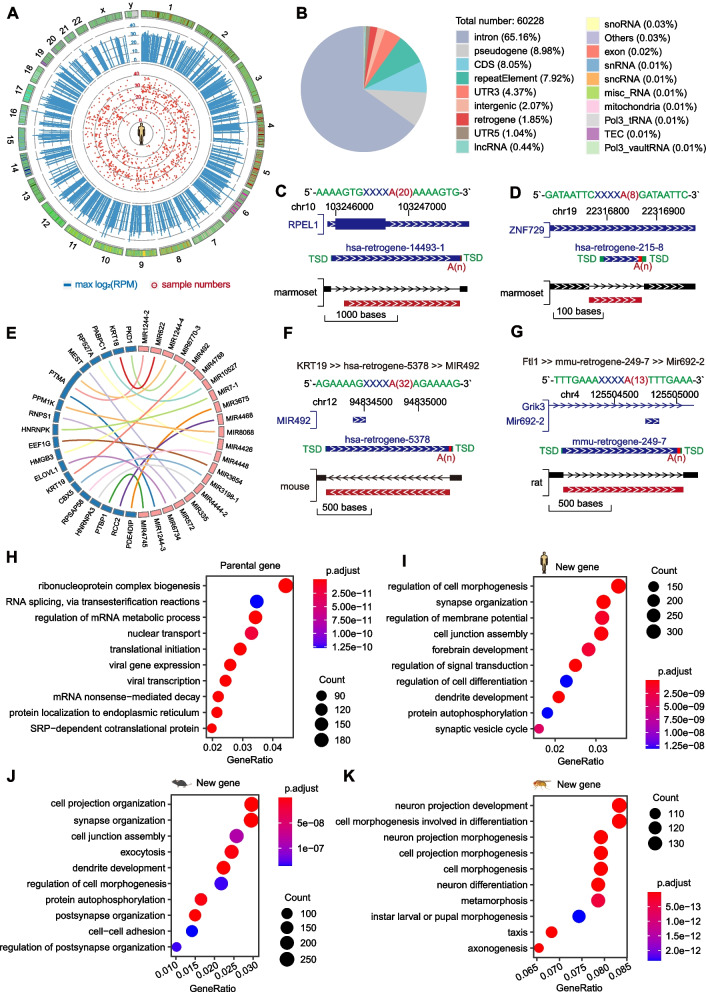
New genes have been generated through retrotransposition. **A** Circos plot illustrating the max log2(RPM) and sample numbers of identified retrotransposons in humans. The top 1000 retrotransposons in terms of expression are shown. The plot legend from the outer circle to the inner circle is shown. Log2(RPM): log2 of the reads per million. **B** Distribution of newly identified human genes in annotated gene types. **C** and **D** Genome Browser visualization for retrotransposons related to RPEL1 (**C**) and ZNF729 (**D**). The whole sequence of the retrotransposon on the top is shown in various colours; green represents TSDs at both termini, blue represents the gene body, and red represents poly(A), of which the number of nucleotides in the poly(A) region is shown in brackets, that is, A(n). The first track shows the information of the known gene annotation. The second track shows the identification result from retroSeeker. The third track partly shows the input net files for retroSeeker. **E** Circos plot illustrating the parent–offspring relationship of retrotransposons related to human miRNA genes. **F** and **G** Genome Browser visualization for retrotransposons related to miRNAs in humans (**F**) and mice (**G**). The whole sequence of the retrotransposon on the top is shown in various colours; green represents TSDs at both termini, blue represents the gene body, and red represents poly(A), of which the number of nucleotides in the poly(A) region is shown in brackets, that is, A(n). The first track shows the information of the known gene annotation. The second track shows the identification result from retroSeeker. The third track partly shows the input net files for retroSeeker. The notation "putative parent gene >  > retrogene >  > miRNA" indicates that the putative parental gene (e.g., KRT19) from another location underwent retrotransposition to the new locus, generating the retrogene (e.g., hsa-retrogene-5378) and finally resulting in the formation of the miRNA (e.g., MIR492). H to K, Top 10 enriched Gene Ontology (GO) biological processes enriched for the parental genes of humans (**H**) and new genes related to the retrotransposons in humans (**I**), mice (**J**) and flies (**K**)

It has been reported that duplication of ancestral genes or domestication of transposable elements could generate new genes during species evolution (Carelli et al. 2016). To rule out potential unexpressed pseudogenes, we required the new gene to exhibit expression of more than 20 unique reads in at least two samples in publicly available RNA-seq datasets from the Encyclopedia of DNA Elements (ENCODE) consortium. We found that a total of 60,228 retrotransposons in humans display the potential to contribute to the creation of newly expressed genes (Fig. 3A and B). Additionally, we identified 25,063 retrotransposons in mice (Figure S3A and C) and 3,385 in Drosophila (Figure S3B and D) that could have similar implications. For instance, a retrotransposition event was responsible for the emergence of the new gene RPEL1 in humans (Fig. 3C), while another retrotransposition event led to the genesis of the new genes Frg2f1 and Uchl4 in mice, which were all included perfect TSDs and long poly(A) (Figure S3E and F). Moreover, retrotransposons contributed indirectly to the creation of new genes by generating new exons. For example, a retrotransposon was found to contribute to the generation of the exons of ZNF729 (Fig. 3D) and POU2F1 (Figure S3G) in humans, allowing for the production of new protein translations.

Interestingly, we detected a group of retrotransposons related to miRNAs originating from protein-coding genes (Fig. 3E). For example, the putative parental gene KRT19 from another location (chr17) underwent retrotransposition to the new locus (chr12), generating hsa-retrogene-5378 and finally resulting in the formation of the miRNA MIR492 (KRT19 >  > hsa-retrogene-5378 >  > MIR492, Fig. 3F). To explore the conserved nature of this miRNA generation mechanism in different species, we performed the same analysis in mice, and we detected a group of retrotransposons related to miRNAs originating from mouse protein-coding genes (Figure S3H). Specifically, a new miRNA, MIR692-2, was generated from the retrotransposons of the protein-coding gene Ftl1 (Ftl1 >  > mmu-retrogene-249–7 >  > Mir692-2, Fig. 3G).

To conduct a preliminary investigation into the functional transition from parental genes to new genes, we traced the parental-to-offspring relationships of each new gene through BLAST searches followed by subsequent Gene Ontology (GO) enrichment analysis. Our analysis revealed that parental genes were involved primarily in ribonucleoprotein complex biogenesis and translation (Fig. 3H), while the new genes were associated mainly with morphogenesis and neuron-related biological processes in humans (Fig. 3I), mice (Fig. 3J) and flies (Fig. 3K). Collectively, these findings strongly indicate that retrotransposition plays a pivotal role in facilitating the birth of novel genes, thereby accelerating the evolutionary journey towards functional diversification.

#### Novel classes of retrotransposons originating from multiple types of genes

**Fig. 4:**
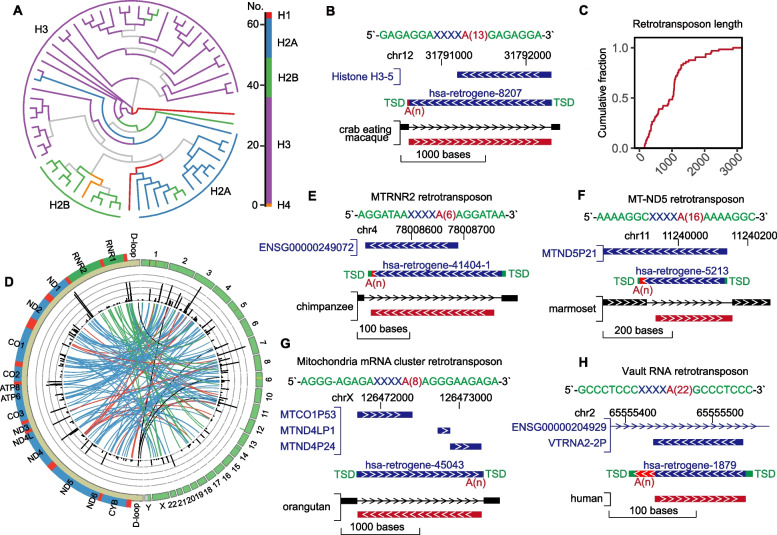
Novel classes of retrotransposons. **A** Phylogenetic tree of retrotransposons from human histone genes. **B** Genome browser visualization for retrotransposons. The whole sequence of the retrotransposon on the top is shown in various colours; green represents TSDs at both termini, blue represents the gene body, and red represents poly(A), of which the number of nucleotides in the poly(A) region is shown in brackets, that is, A(n). The first track shows the information of the known gene annotation. The second track shows the identification result from retroSeeker. The third track partly shows the input net files for retroSeeker. **C** Cumulative curves showing the lengths of retrotransposons from human histone genes. **D** Circos plot illustrating the parent–offspring relationship of retrotransposons related to human mitochondrial genes. **E** to **H** Genome Browser visualization for retrotransposons related to mitochondrial genes, including rRNA (**E**), mRNA (**F**), and mRNA cluster (**G**), and related to the vault RNA gene (**H**). The whole sequence of the retrotransposon on the top is shown in various colours; green represents TSDs at both termini, blue represents the gene body, and red represents poly(A), of which the number of nucleotides in the poly(A) region is shown in brackets, that is, A(n). The first track shows the information of the known gene annotation. The second track shows the identification result from retroSeeker. The third track partly shows the input net files for retroSeeker

To further discover the novel classes of retrotransposons, we focused on the gene types of candidate retrotransposons that were previously uncharacterized. Notably, we detected a group of histone genes that generated new copies through retrotransposition. In humans (Fig. 4A) and mice (Figure S4A), these copies were primarily H3, while in flies, they were mainly from H2B (Figure S4B). For instance, a new copy of H3-5 was generated from H5 through retrotransposition in humans, and it was observed to be longer than the parental copy (Fig. 4B). To further characterize the retrotransposons originating from histone genes, we conducted a statistical analysis on their lengths and found that they ranged mainly from 1000 to 3000 nt (Fig. 4C).

Our investigation also revealed that mitochondrial RNAs might integrate into the nuclear genome through retrotransposition (Fig. 4D and Figure S4C). For instance, a putative mitochondrial rRNA retrotransposition was found to be embedded in chromosome 4 in humans (Fig. 4E). Additionally, a predicted mitochondrial mRNA retrotransposition was embedded in an intergenic region of chromosome 11 (Fig. 4F), and a putative mitochondrial mRNA retrotransposition containing three parental genes was embedded in the intergenic region of chromosome X (Fig. 4G) with a standard poly(A) region and TSD sequences.

Finally, we found that the noncoding vault RNAs were innovative origins for creating an entirely new class of retrotransposons (Fig. 4H). For example, we identified a retrotransposon of vault RNAs with perfect TSDs and a poly(A) region in humans (Fig. 4H). Together, these results suggest that histone genes, mitochondrial RNAs, and other ncRNA genes may serve as novel sources to generate novel retrotransposon categories by a shared L1-mediated reverse transcription mechanism.

#### Atlas of transcriptionally active retrotransposons

**Fig. 5:**
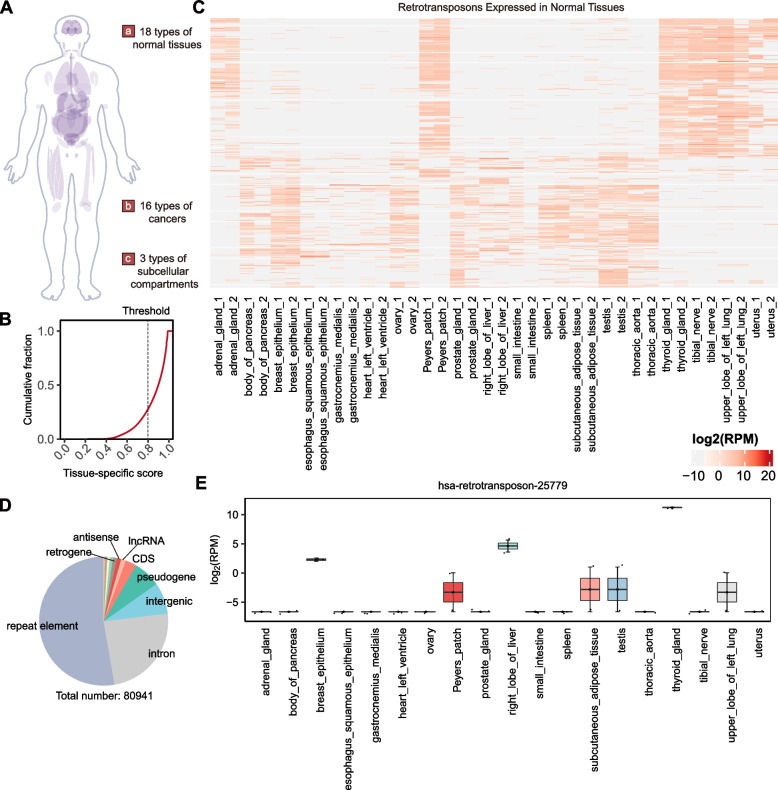
Atlas of tissue-specific retrotransposons. **A** Schematic depiction of the collection of three types of expression datasets. **B** Cumulative curves showing the tissue-specific Tau scores of all retrotransposons identified in humans. **C** Heatmap showing the expression profiles of retrotransposons in various human tissues using total RNA-seq data from ENCODE. The expression levels in cells were categorized into corresponding tissues. log2RPM: log2 of the reads per million. **D** Distribution of identified tissue-specific retrotransposons in annotated gene types. **E** Expression values of a representative tissue-specific retrotransposon, hsa-retrotransposon-25779. Box plot showing the log2RPM (RPM, reads per million) values in different tissues. Each box shows the first quartile, median, and third quartile

To comprehensively examine the tissue-specific patterns of retrotransposons, we further analysed their expression profiles across nineteen types of normal tissue using publicly available RNA-seq datasets produced by the ENCODE consortium (Fig. 5A). We used tau score arithmetic to calculate the tissue specificity of the retrotransposons and found that numerous retrotransposons exhibited distinct expression patterns in specific human tissues (Fig. 5B and C). In total, we identified 80,941 tissue-specific retrotransposons with a Tau score higher than 0.8 in humans, predominantly located within repetitive elements, introns, and intergenic regions across the human genome (Fig. 5D). For example, hsa-retrotransposon-25779 exhibited a significantly higher expression level in the thyroid gland than in other tissues (Fig. 5E). To explore the tumour profile of the retrotransposons, we analysed the expression of retrotransposons using RNA sequencing data from 16 types of cancers obtained from the Cancer Cell Line Encyclopedia (CCLE). Interestingly, we found that a subset of retrotransposons exhibited ubiquitous expression across various types of cancers without any cancer type-specific patterns (Figure S5A).

To investigate the distribution of expressed retrotransposons in subcellular RNA fractions, we analysed the subcellular expression data and observed that most retrotransposons displayed nuclear-specific localization patterns, with higher expression levels in the nucleoplasm and nucleolus than in the cytoplasm (Figure S5B).

To explore the tissue specificity of the retrotransposons in other representative species, we further analysed the expression data from mice and flies. We detected a subset of tissue-specific retrotransposons in mice (Figure S5C) and flies (Figure S5D). Remarkably, we found that a large number of retrotransposons were highly expressed in the midbrains of mice (Figure S5C). Together, our findings suggest that a large number of retrotransposons display specific expression patterns in certain tissues while exhibiting ubiquitous expression in cancers.

#### Complex evolution patterns of retrotransposons across multiple species

**Fig. 6:**
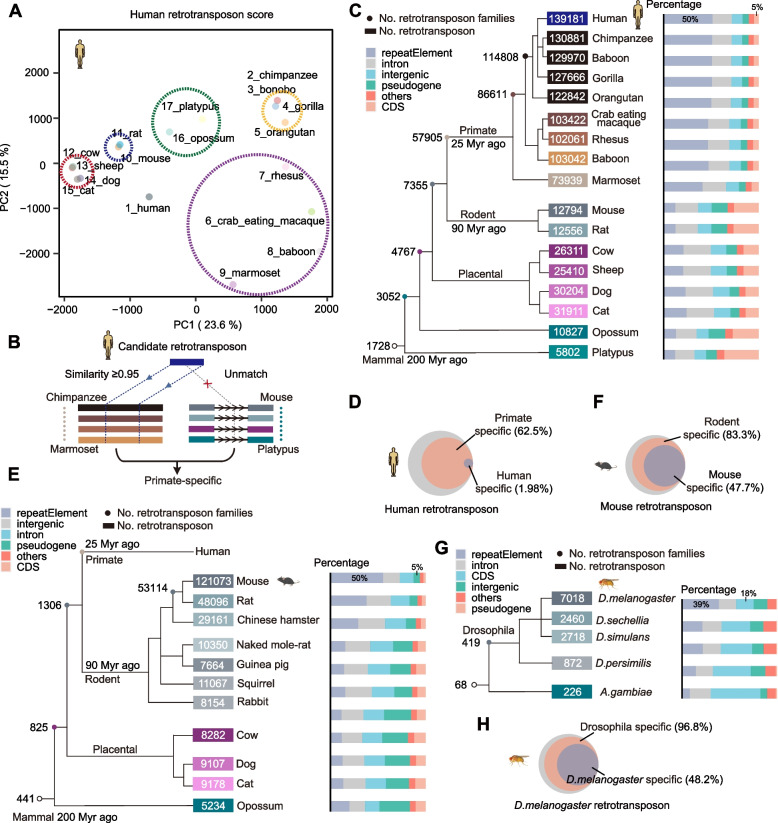
Complex evolution patterns of retrotransposons. **A** Principal component analysis (PCA) of the retrotransposon scores in multiple species. **B**, Schematic depiction of the identification of species-specific retrotransposons. **C** Simplified phylogenetic trees of human retrotransposons. Internal branches and roots, numbers of orthologous retrotransposon families for the indicated species. Tree tips, retrotransposon numbers for each species. **D** Venn diagram depicting the number of unique and shared items that were identified in total, in primates, and in humans. **E** Simplified phylogenetic trees of mouse retrotransposons. Internal branches and roots, numbers of orthologous retrotransposon families for the indicated species. Tree tips, retrotransposon numbers for each species. **F** Venn diagram depicting the number of unique and shared items that were identified in total, in rodents, and in mice. **G** Simplified phylogenetic trees of fly retrotransposons. Internal branches and roots, numbers of orthologous retrotransposon families for the indicated species. Tree tips, retrotransposon numbers for each species. **H** Venn diagram depicting the number of unique and shared items that were identified in total, in Drosophila, and in flies

To investigate the evolutionary relationships of retrotransposons identified from various species, we performed principal component analysis (PCA) on the retrotransposon scores across multiple species. Remarkably, we observed that the retrotransposons could be classified into five clades, aligning neatly with known species phylogenesis (Fig. 6A). To explore the representative evolutionary profiles of different types of retrotransposons, we first conducted a statistical analysis of the composition of Alu subfamilies (Figure S2A). We found that the AluS subfamily occupies the largest proportion (60%, Figure S6A), which is known to be the second oldest subfamily within the Alu elements. Specifically, the AluY (4400), AluSx (2161) and AluSx1 (2086) subfamilies emerged as the most successful retrotransposons through retrotransposon expansion (Figure S6B). Here, we presented several examples in Alu subfamilies that exhibit perfect TSDs and canonical poly(A) regions (Figure S6C, D and E).

To systematically explore species-specific retrotransposons and further investigate their evolutionary pattern, we mapped the sequences of the candidate retrotransposons to the genomes of other species with a requirement of a sequence similarity ≥ 0.95 (Fig. 6B). We found that a significant proportion of retrotransposition events occurred (7355 to 57,905) after the differentiation of primates approximately 25 Myr ago (Fig. 6C, left panel).

To further explore the genomic distribution of retrotransposons across species evolution, we mapped the retrotransposon loci to known gene annotations within various species individually. Interestingly, we found that throughout species evolution, the proportion of repetitive elements increased to approximately 50%, while the proportion of coding sequences (CDSs) decreased to approximately 5% (Fig. 6C, right panel). We discovered that more than half of the retrotransposons in humans were primate-specific (62.5%), but only a minor proportion were specific to humans (1.98%, Fig. 6D). Interestingly, we also detected retrotransposons that likely originated over 200 million years ago (Myr) (Fig. 6C).

To explore the evolutionary patterns in other model organisms, we extended the analysis to mice and flies. We observed that the majority of retrotransposition occurred (53,114 to 121,073) after the differentiation of mice (Fig. 6E, left panel). Moreover, the proportion of repetitive elements increased to approximately 50%, while the proportion of pseudogenes decreased to approximately 5% (Fig. 6E, right panel). Our investigation revealed that 62.5% of retrotransposons were exclusive to rodents, with 47.7% specifically identified in mice (Fig. 6F). Interestingly, nearly all retrotransposons identified in flies belonged to the Drosophila genus (96.8%) (Fig. 6G and H). Together, our findings suggest that the dynamic rearrangement of retrotransposon composition adds to the complexity and diversity of species genomes.

## Discussions

By mapping the biological model (copy and paste) of retrotransposition events into comparative genomics data (gaps and fills), retroSeeker is capable of accurately identifying new retrotransposons in the genomes of any species. We found an increased diversity of retrotransposons dispersed throughout coding/noncoding genes and intergenic regions (Figure S2A, B and C) through a conserved recognition and insertion mechanism. We observed that a large proportion of retrotransposons identified in humans and mice have several consensus sequence elements located around the 5’-ends of retrotransposons (Fig. 2K, L, S2F and G). Importantly, one of the consensus sequence elements occurs frequently ten nucleotides upstream of the retrotransposon insertion sites, forming a site-specific TTAAAAN_(10)_ motif (Fig. 2M). Interestingly, we did not detect these motifs in flies, suggesting that this mechanism may have arisen after mammalian differentiation and promoted retrotransposon amplification in higher organisms. Although site-specific motifs have also been reported in silkworm R2 retrotransposons, they target only the 28S rRNA gene in vivo (Wilkinson et al. 2023). In contrast to R2 retrotransposons with strict restriction, ORF2-based retrotransposons target a wider range of gene types throughout the entire genome (Figure S2A and B). This discovery suggests that the ORF2 protein could be a potential tool for in vivo gene editing.

Understanding how new genes originate is crucial for explaining the genetic basis for the origin and evolution of novel phenotypes and biological diversity. In contrast to previous studies focused on limited mRNA-derived gene duplicates, we systematically explored all genes related to retrotransposition. Our work suggests that there are two major pathways for the formation of new genes through retrotransposition (Fig. 3B). The first pathway involves the duplication of a parental gene by retrotransposition followed by subsequent mutation or neofunctionalization processes (Fig. 3C). The second pathway involves the insertion of these retrocopies into preexisting genes, resulting in a chimeric RNA product that combines retrotransposons with gene sequences (Fig. 3D). In contrast to the parental genes that are primarily involved in ribonucleoprotein complex biogenesis and translation (Fig. 3H), we observed that the new genes generated by retrotransposition primarily participate in various cellular development and differentiation processes (Fig. 3I), indicating the expansion of their functional repertoires. This observation is consistent with recent work highlighting how orphan retrogenes functionally replace their parental genes (Carelli et al. 2016). For example, human POU2F1, a new gene shaped by retrotransposition (Figure S3G), is a transcription regulator in higher eukaryotes that is involved in the regulation of development, differentiation, stress responses and other processes (Hamashima et al. 2023).

It has been proposed that the number and polymorphism of retrotransposition-active ncRNAs may be comparable to those of active Alu or L1 elements (Weber 2006). However, this question is largely unknown due to the lack of a complete list for ncRNA retrotransposons. Thus, we conducted, for the first time, a comprehensive investigation into the entire range of RNA species. Importantly, we observed vault RNAs have retrotransposition ability in humans (Fig. 4H), illuminating a conserved mechanism of generating functional copies for ncRNA genes in mammals. Interestingly, we detected the putative embedding of mitochondrial (MT) RNAs into the nuclear (NU) genome (Fig. 4D and C), generating nuclear-mitochondrial segments (NUMTs). NUMTs are present throughout the entire human nuclear genome, and some NUMTs are associated with disease (Xue et al. 2023). In contrast to the previously proposed mechanism in which NUMTs are integrated by double-stranded DNA break repair (Wei et al. 2022), our work reveals a novel optional pathway for NUMT generation. However, how mtDNA/mtRNA fragments exit the mitochondrion and translocate into the nucleus is not known (Xue et al. 2023); therefore, future studies should conduct more effective validation.

Retrotransposons were once considered “genomic junk” that were not expressed due to a lack of transcription elements. However, more recent works have highlighted their substantial regulatory role in tissue differentiation (Roller et al. 2021; Nam et al. 2023). In this study, we systematically identified a large set of retrotransposons with tissue-specific expression patterns, shedding light on their roles in gene expression regulation. We detected a significant proportion of retrotransposons embedded within intronic or intergenic regions (Fig. 5D). Thus, we hypothesize that these retrotransposons might contribute to new exons or tissue specificity by generating novel promoters, enhancers or other regulatory elements (e.g. enhancer RNAs) to shape tissue-specific gene regulatory patterns. Intriguingly, we also observed prominent high expression of a large set of retrotransposons in the brain (Figure S5C), which is consistent with recent work highlighting retrotransposons as important drivers of mammalian brain evolution (Ferrari et al. 2021). Moreover, dysregulation of retrotransposon activity may also contribute to neurological disease. Therefore, we hypothesized that retrotransposons with specificity in other tissues might also play important roles in other diseases.

In summary, our study provides valuable software and reveals retrotransposon diversity within the genomes of humans, mice and flies. The findings described here contribute to the understanding of the biogenesis mechanisms of retrotransposons and introduce hundreds of thousands of novel retrotransposons whose functions can now be investigated. Further applications of retroSeeker to additional populations and organisms will deepen our understanding of retrotransposon complexity.

## Methods

### Identification of retrotransposons from pairwise alignment data

Pairwise genome alignments of multiple species were downloaded from the UCSC Genome Browser website (Navarro Gonzalez et al. 2021) in the net file format, with the three central species being human, mouse, and fly. For a detailed description of the net file format, refer to the UCSC Genome Browser description pages (https://genome.ucsc.edu/goldenPath/help/net.html) and the download page (https://hgdownload.soe.ucsc.edu/goldenPath/hg38/vsMm10/) of the net file format. In addition, pairwise genome alignment files (.net) can also be generated through our open source pipeline (https://github.com/junhong-huang/retroSeeker/make_pairwise_alignment_pipeline.pl), which includes the following steps: (1) generation of the pairwise genome alignments in axt format by lastz software (https://github.com/lastz/lastz) with the parameters “–format = axt –ambiguous = iupac ‑‑action:target = multiple –strand = both –allocate:traceback = 1.99G”, (2) conversion of the axt file format (.axt) into the chain format (.chain) by axtChain software (http://hgdownload.soe.ucsc.edu/admin/exe/linux.x86_64/axtChain) with the parameter “-linearGap = loose”, and (3) conversion of the chain file format to the net format (.net) by chainNet software (http://hgdownload.soe.ucsc.edu/admin/exe/linux.x86_64/chainNet) with the default parameters.

Subsequently, we utilized retroSeeker, taking the net-format file as input, to identify potential retrotransposons. The "self mode" was employed for the same species genome (e.g., human–human net), while the "different species mode" was utilized for other species (e.g., human–mouse net), with the default parameters. By mapping the biological concepts (copy and paste) of retrotransposition events into comparative genomics data with net format (gaps and fills) (Weber 2006), retroSeeker can identify retrotransposons in the genomes of any species. The detailed workflow is as follows: (1) Search for the gap region within the query genome (i.e., the region present in the reference genome but not in the query genome). (2) Search for the fill region within the aforementioned gap region (i.e., the region present in both the reference and query genomes). (3) Extend a certain length of nucleotides inwards and outwards at the upstream and downstream ends of the fill region, respectively. (4) Employ dynamic programming algorithms to identify TSDs that are at least seven nucleotides in length. (5) Search for the poly(A) region adjacent to the 3'-TSD. (6) Obtain the reverse complementary sequence of the fill region and repeat steps 4 and 5. (7) Evaluate the poly(A) and TSD features using a scoring system and retain retrotransposon candidates with higher scores. To identify high-confidence candidates, we only included retrotransposons with TSD length ≥ 7 and poly(A) length ≥ 5 for further analysis.

### Simulation studies to assess sensitivity and specificity

To evaluate the sensitivity and specificity of retroSeeker, we conducted a simulation study using a computer-generated genome consisting of 60,000,000 random bases of A/T/C/G. Initially, we simulated the parent of the LINE1 retrotransposon by randomly incorporating one original LINE1 sequence into the genome. Then, the sequence of LINE1 was randomly modified to include specific retrotransposon features, such as poly(A) tail lengths ranging from 5 to 20 nucleotides, target site duplications (TSDs) of 7 to 20 nucleotides with random bases of A/T/C/G, and a plus- or minus-strand orientation of insertion. We generated a total of 1000 retrotransposons, with each one being randomly integrated into the simulated genome. As a control, we also inserted 1000 original LINE1 sequences without TSDs and poly(A) tails. Finally, pairwise alignment of the whole genome before and after the random insertion of retrotransposon sequences was performed using lastz with the parameters “–format = axt –ambiguous = iupac ‑‑action:target = multiple –strand = both –allocate:traceback = 1.99G”. Moreover, we performed a ROC (receiver operating characteristic curve) analysis on the retrotransposon scores generated by retroSeeker using the R package pROC (Robin et al. 2011). This analysis allowed us to evaluate the performance of retroSeeker in distinguishing retrotransposons from nonretrotransposon sequences.

### Annotation and visualization of retrotransposons

The genome sequences of humans (hg38), mice (mm10) and flies (dm6) were downloaded from the UCSC Genome Browser website. Human and mouse gene annotations were acquired from GENCODE (human release 39 and mouse release 25) (Frankish et al. 2021). The fly gene annotations were acquired from UCSC (ensGeneV101). The repetitive elements identified by RepeatMasker were downloaded from the UCSC Genome Browser website. All retrotransposons were intersected with canonical gene annotations using BEDTools software (Quinlan and Hall 2010). Individual retrotransposons were visualized with the trackBrowser tool (Zhang et al. 2023), and the genomic distribution of retrotransposons and their relations to the parental genes were visualized with Circos (Krzywinski et al. 2009).

### Motif analysis and visualization

Highly similar sequences were clustered using CD-HIT (Fu et al. 2012) with the parameter settings "-M 0 -c 0.80 -d 150 -T 30 -s 0.8 -A 0.8 -sc 1". Subsequently, only one representative sequence was retained prior to motif detection. We de novo-identified motifs using the script findMotifsGenome.pl of HOMER (Heinz et al. 2010) with the parameters “-norevopp -noknown -rna -len 4,5,6,7,8,9 -p 20 -size given -dumpFasta” and MEME with the parameters “-rna -minw 5 -maxw 50 -allw -maxsize 0 -nmotifs 50 -brief 20,000 -p 10 -evt 5”.

### Analysis of the evolution and putative functions of retrotransposons

The sequences of retrotransposons related to histone genes were aligned using ClustalW 2.1 (Chenna et al. 2003) with the default parameters. Phylogenetic trees were constructed with FastTree 2.1 (Price et al. 2010) with the default parameters and plotted with the R package ggtree (Yu 2020). GO analysis was performed with clusterProfiler (Yu et al. 2012), and only the GO terms with similarity less than 0.7 were retained.

### Transcriptome-wide data analyses of retrotransposons

RNA-seq data of tissues were download from ENCODE consortium (Consortium, Moore et al. 2020), and RNA-seq data of cancers were download from CCLE (Barretina et al. 2012). The clean reads were mapped to the reference genome (hg38) with STAR (Dobin et al. 2013) software with the following parameters: –outSAMmultNmax 1 –outFilterMultimapNmax 1 –genomeLoad NoSharedMemory –alignEndsType Local –outFilterType Normal –outFilterMultimapScoreRange 0 –outFilterMismatchNmax 15 –outFilterMismatchNoverLmax 0.1 –outFilterScoreMin 0 –outFilterScoreMinOverLread 0 –outFilterMatchNmin 18 –outFilterMatchNminOverLread 0.8 –alignIntronMin 5 –seedSearchStartLmax 15 –seedSearchStartLmaxOverLread 1 –seedSearchLmax 0 –alignTranscriptsPerReadNmax 20,000 –alignWindowsPerReadNmax 20,000 –seedMultimapNmax 20,000 –seedPerReadNmax 1000 –seedPerWindowNmax 100 –seedNoneLociPerWindow 20 –outSAMtype BAM Unsorted –outSAMmode Full –outSAMattributes All –outSAMunmapped None –outSAMorder Paired –outSAMprimaryFlag AllBestScore –outSAMreadID Standard –outReadsUnmapped Fastx –scoreGapNoncan -8 –scoreGapATAC -8 –scoreGapGCAG -4 –alignSJoverhangMin 15 –alignSJDBoverhangMin 5 –alignEndsProtrude 150 ConcordantPair –scoreGenomicLengthLog2scale -1 –readFilesCommand zcat. Then featurecounts (Liao et al. 2014) was used to calculate the expression of the retrotransposons region using parameters “-F SAF -s 1 -T 16 -p”.

### Tissue-specific retrotransposon identification

As a measure for tissue specificity, we used the Tau score, the best choice for calculating tissue specificity among existing methods (Kryuchkova-Mostacci and Robinson-Rechavi 2017), which is calculated on the basis of the log2 RNA-seq expression data. The values of Tau vary from 0 to 1, where 0 indicates ubiquitous expression and 1 indicates specific expression. Genes with Tau values greater than 0.8 were considered tissue specific (Kryuchkova-Mostacci and Robinson-Rechavi 2016).

## Supplementary Information

**Additional file 1. Figure S1. **A new computational approach for the discovery of retrotransposons. **Figure S2.** Characteristics of retrotransposons. **Figure S3.** New genes were generated through retrotransposition. **Figure S4.** Novel classes of retrotransposons.** Figure S5.** Atlas of tissue-specific retrotransposons. **Figure S6.** The complex evolution patterns of retrotransposons.

**Additional file 2: Supplementary Table 1. **Species used in this study.

**Additional file 3: Supplemental Table S2. **Retrotransposons identified in human.

**Additional file 4: Supplemental Table S3. **Retrotransposons identified in mouse.

**Additional file 5: Supplemental Table S4. **Retrotransposons identified in fly.

## Data Availability

All data supporting the findings of this study can be found within the paper. Additional data supporting the findings of this study are available from the corresponding author upon request.
