# Supplementary material for: RetroSeeker reveals the characteristics, expression, and evolution of a large set of novel retrotransposons
Source: Adv Biotechnol (Singap). 2023 Oct 31;1(4):5. doi: 10.1007/s44307-023-00005-5 (PMC11727581; doi:10.1007/s44307-023-00005-5)
Supplement: Supplementary file 1 — Additional file 1. Figure S1. A new computational approach for the discovery of retrotransposons. Figure S2. Characteristics of retrotransposons. Figure S3. New genes were generated through retrotransposition. Figure S4. Novel classes of retrotransposons. Figure S5. Atlas of tissue-specific retrotransposons. Figure S6. The complex evolution patterns of retrotransposons. [file 44307_2023_5_MOESM1_ESM.pdf]

Supplementary information for

RetroSeeker Reveals the Characteristics, Expression, and Evolution of a Large Set of Novel Retrotransposons

Authors: Junhong Huang[1,2], Zhirong Chen[1,2], Bin Li[1], Lianghu Qu[1,†], Jianhua Yang[1,2,†]

Correspondence to: Jianhua Yang (Email: yangjh7@mail.sysu.edu.cn), Lianghu Qu (Email: lssqlh@mail.sysu.edu.cn)

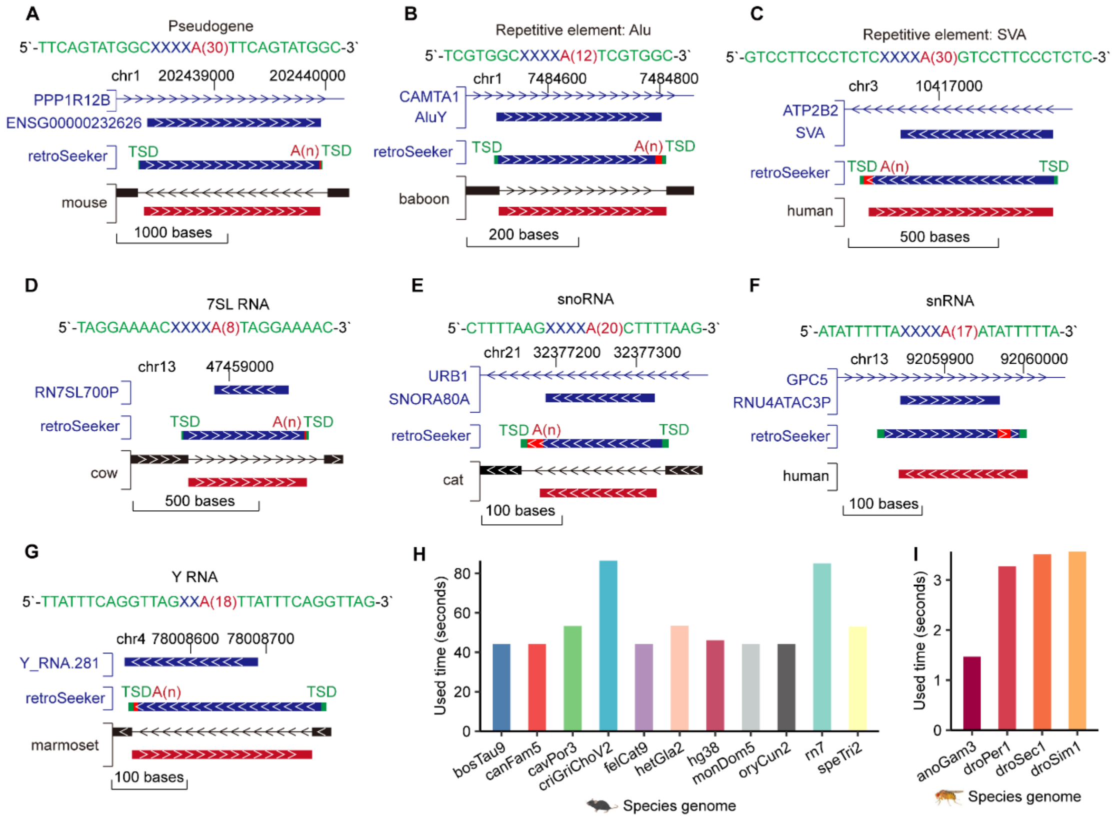

Figure S1. A new computational approach for the discovery of retrotransposons. A to G, Genome Browser visualization for retrotransposon related to pseudogene (A), Alu (B), SVA (C), 7SL RNA (D), snoRNA (E), snRNA (F) and Y RNA (G). The whole sequence of retrotransposon on the top was shown in various colours, green represents TSDs at both termini, blue represents gene body and red represents poly(A), of which the number of nucleotides in the poly(A) region were shown in the bracket, that is, A(n). The first track showed the information of the known gene annotation. The second track showed the identification result from retroSeeker. The third track partly showed the input net files for retroSeeker. **H and I**, The used time of retroSeeker for different input net files based on mouse (H) and flies (I). Genome version of net files were shown.

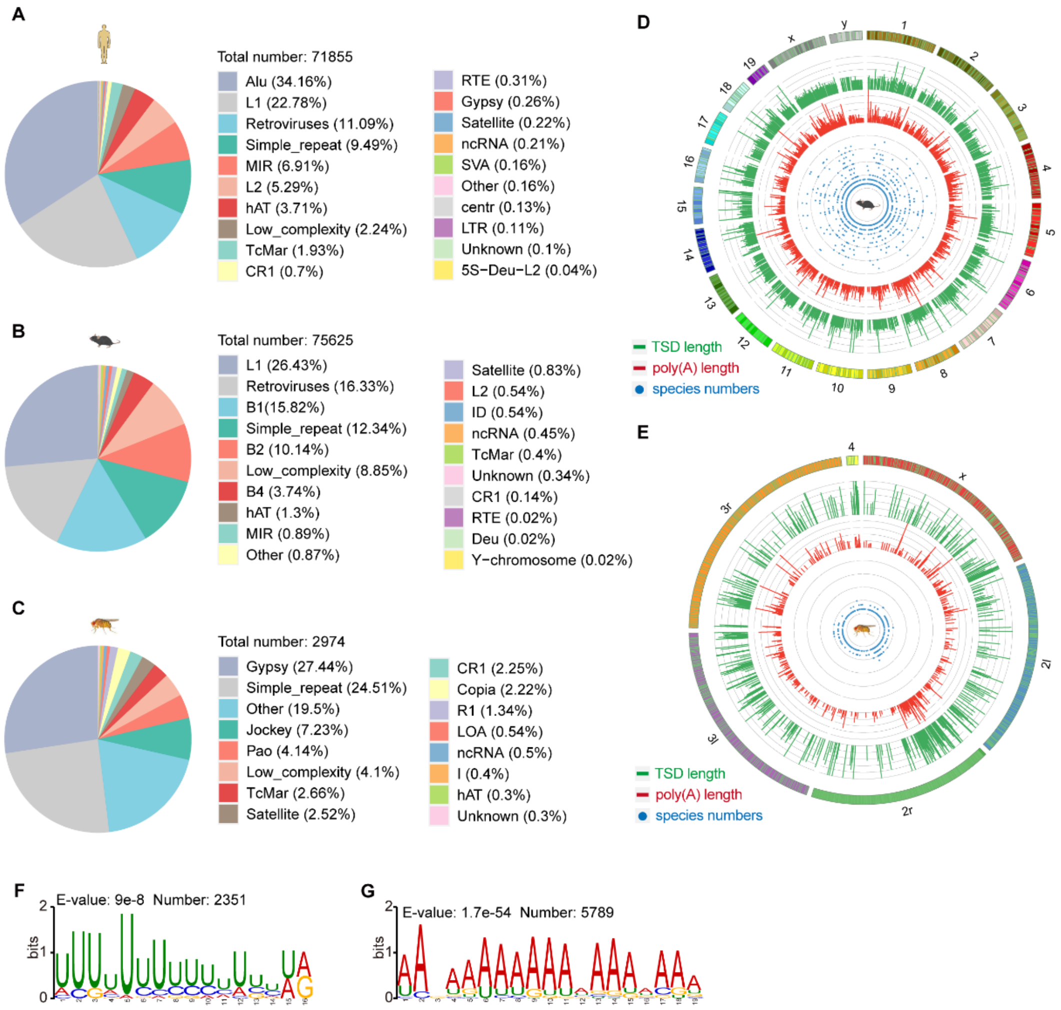

Figure S2. Characteristics of retrotransposons. A to C, Distribution of identified retrotransposon of human (A), mouse (B) and flies (C) in annotated repetitive element types. **D and E**, Circos plot illustrating the TSD length, poly(A) length and species numbers of identified retrotransposon in mouse (D) and flies (E). The plot legend from outer circle to inner circle is shown. **F and G**, Sequence motif obtained within the upstream and downstream 20nt sequences of 5`-start sites of the retrotransposons in human.

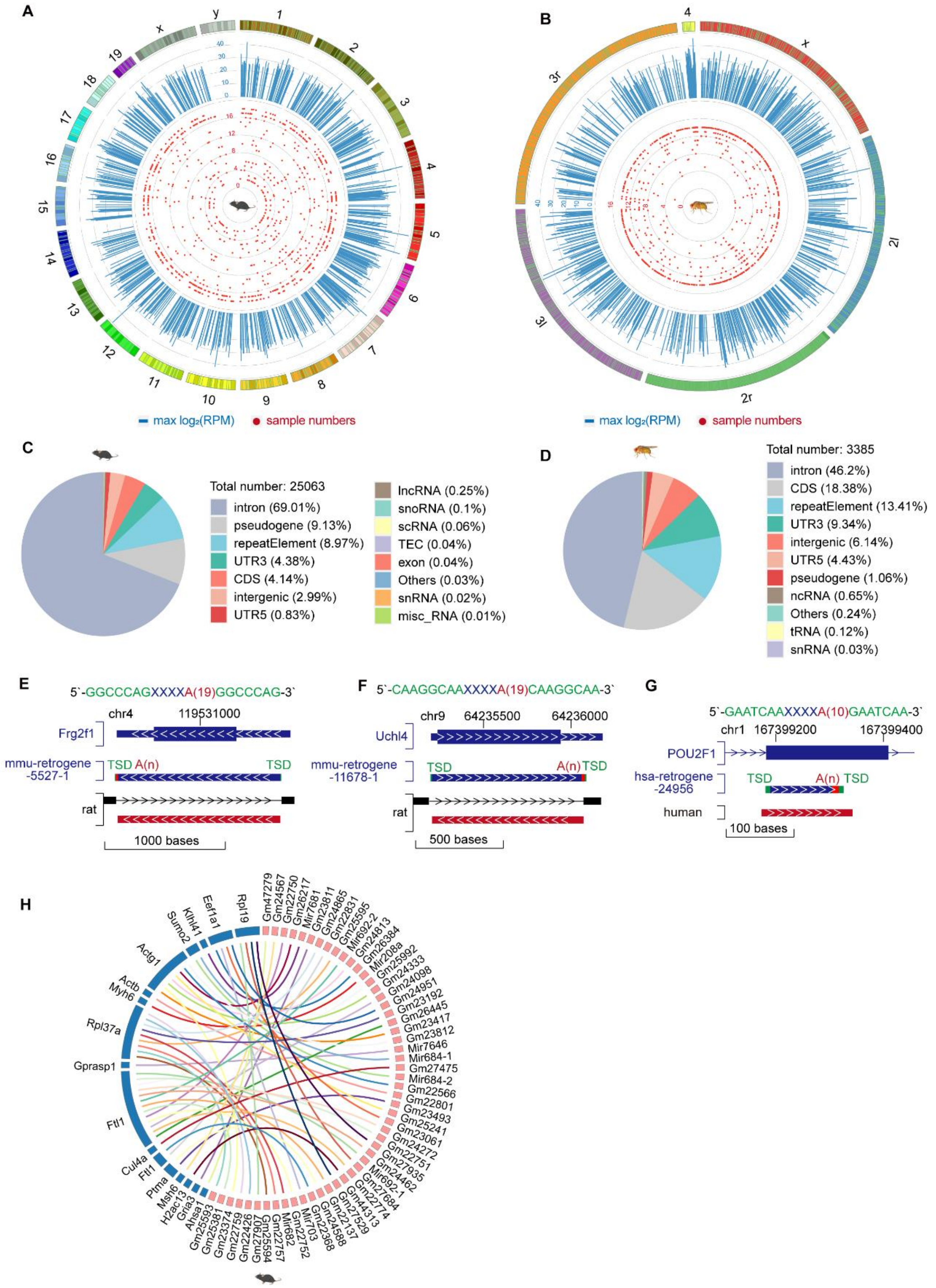

Figure S3. **New genes were generated through retrotransposition. A and B** Circos plot illustrating the max log2(RPM) and sample numbers of identified retrotransposons in mouse (A) and flies (B). Top 1000 retrotransposons in expression were shown. The plot legend from outer circle to inner circle is shown. log2(RPM): log2 for reads per million. **C and D**, Distribution of identified new genes of mouse (C) and flies (D) in annotated gene types. **E to G**, Genome Browser visualization for retrotransposon related to Frg2f1 (E), Uchl4 (F) and POU2F1 (G). The whole sequence of

retrotransposon on the top was shown in various colours, green represents TSDs at both termini, blue represents gene body and red represents poly(A), of which the number of nucleotides in the poly(A) region were shown in the bracket, that is, A(n). The first track showed the information of the known gene annotation. The second track showed the identification result from retroSeeker. The third track partly showed the input net files for retroSeeker. **H**, Circos plot illustrating the parent-spring relationship of retrotransposons related to mouse miRNA genes.

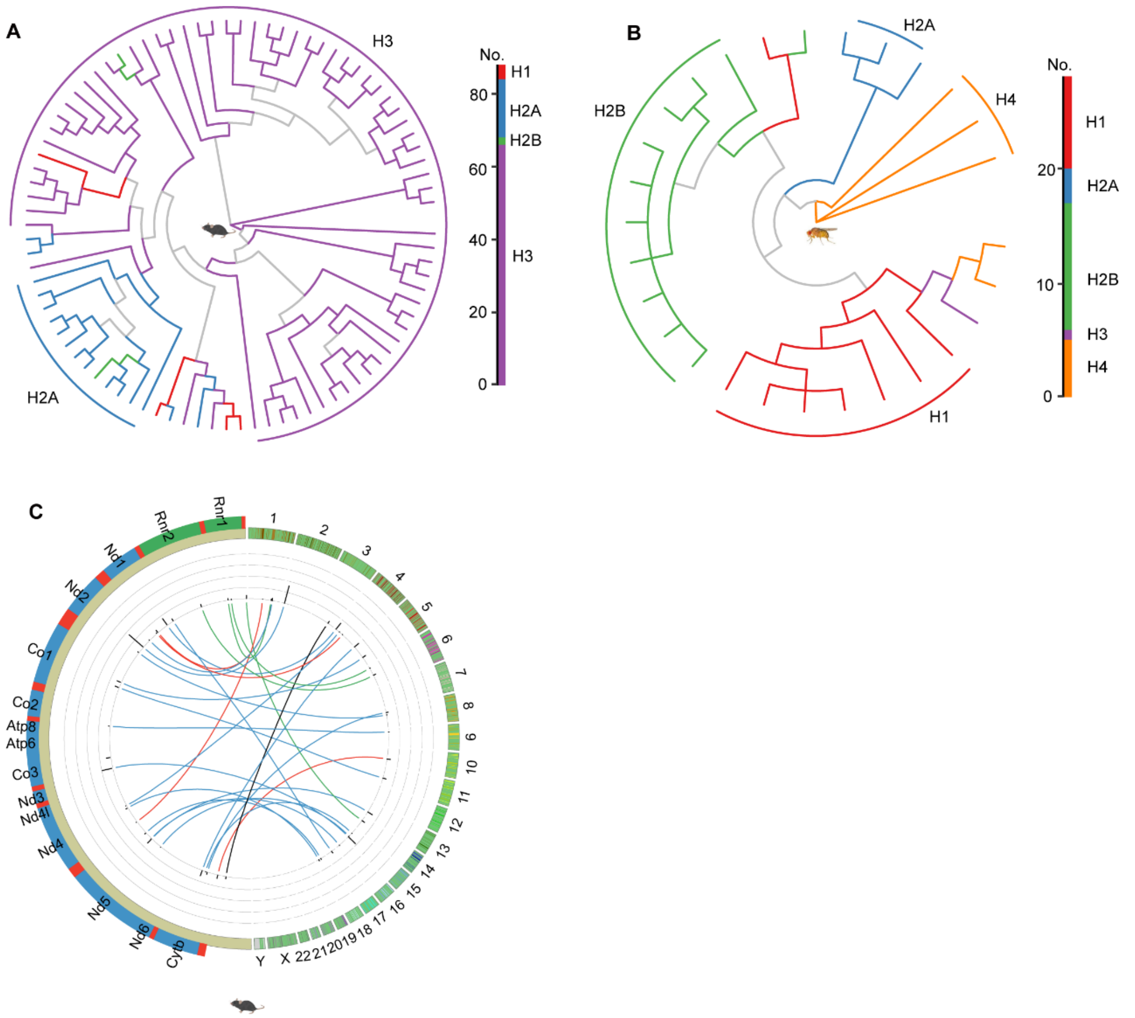

Figure S4. Novel classes of retrotransposons. A and B, Phylogenetic tree of retrotransposons from mouse (A) and flies (B) histone genes. **C**, Circos plot illustrating the parent-spring relationship of retrotransposons related to mouse mitochondria genes.

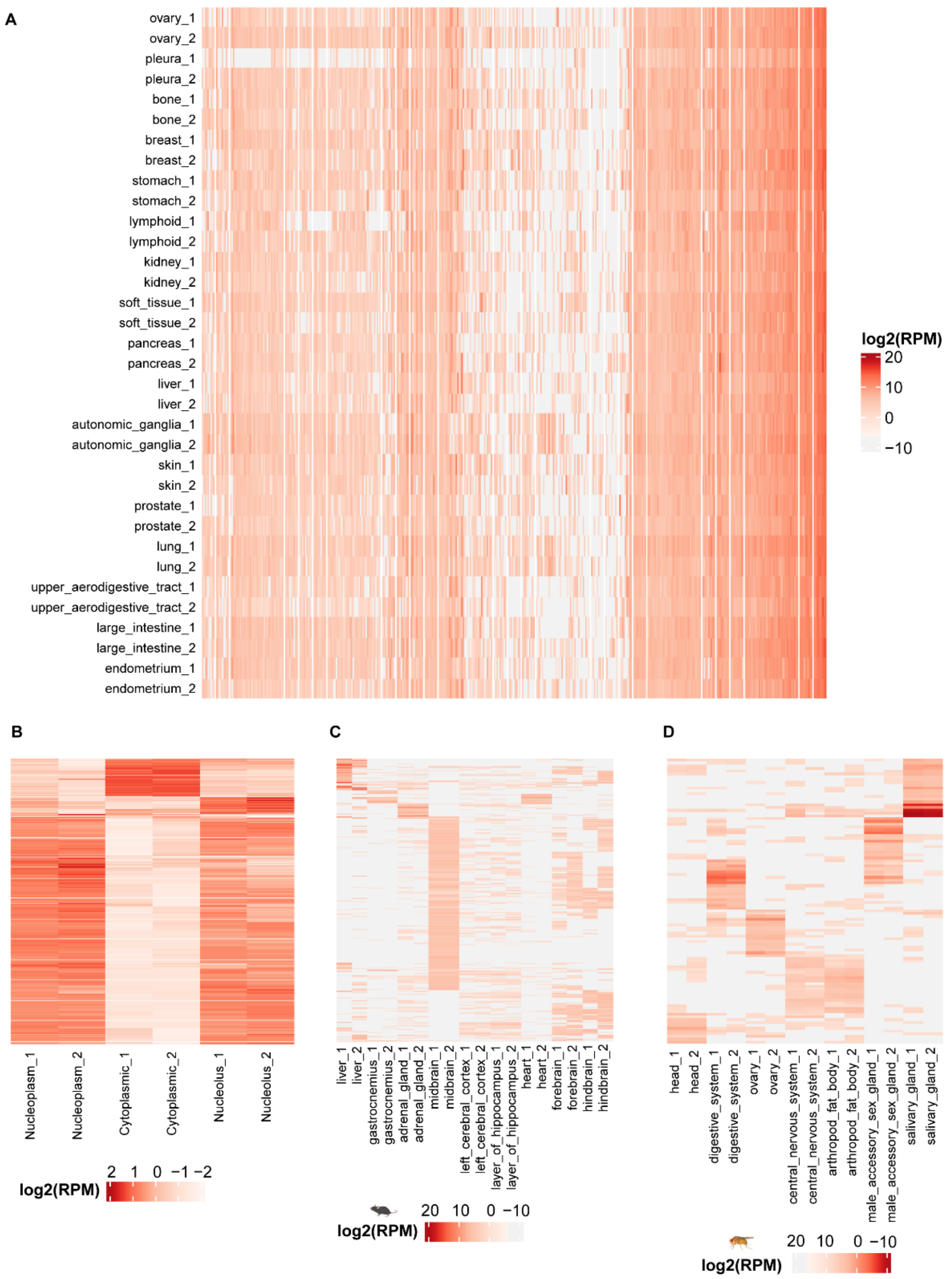

Figure S5. Atlas of tissue-specific retrotransposons. A, Heatmap showing the expression profiles of retrotransposons in various metastasis cancers using RNA-seq data from CCLE. The expression levels in cells were categorized into corresponding cancers. log2RPM: log2 for reads per million. **B** Heatmap showing the expression profiles of retrotransposons in different subcellular regions in cytoplasm, nucleoplasm and Nucleolus of k562 cells. **C and D,** Heatmap showing the expression profiles of retrotransposons in various mouse (C) and flies (D) tissues using total RNA-seq data from ENCODE. The expression levels in cells were categorized into corresponding tissues. log2RPM: log2 for reads per million.

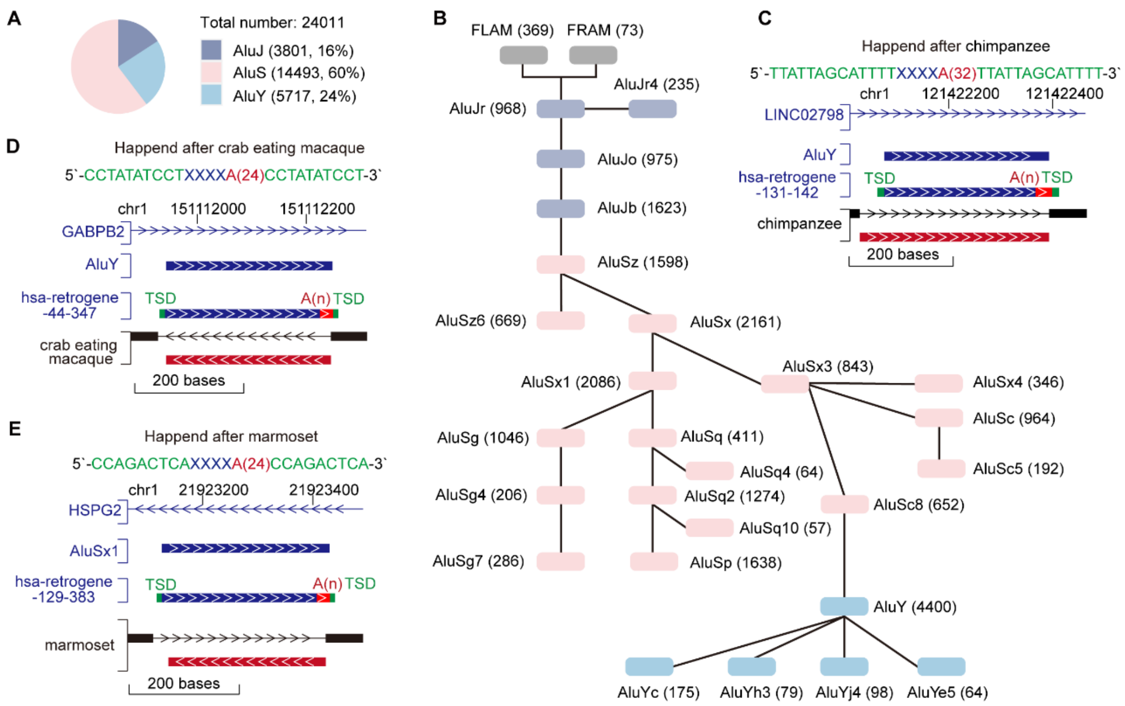

Figure S6. **The complex evolution patterns of retrotransposons. A,** Distribution of identified retrotransposons of human in annotated Alu types. **B,** Simplified phylogenetic trees of human Alu repetitive element. The number within bracket represents the number of specific subclass of retrotransposons. **C to E,** Genome Browser visualization for retrotransposon identified through chimpanzee (C), crab eating macaque (D), marmoset (E). The whole sequence of retrotransposon on the top was shown in various colours, green represents TSDs at both termini, blue represents gene body and red represents poly(A), of which the number of nucleotides in the poly(A) region were shown in the bracket, that is, A(n). The first track showed the information of the known gene annotation. The second track showed the identification result from retroSeeker. The third track partly showed the input net files for retroSeeker.
